# Old Master Zhu: in memory of virologist Guan-Fu Zhu

**DOI:** 10.1007/s13238-017-0396-4

**Published:** 2017-03-18

**Authors:** Qing Ye, Tao Jiang, Cheng-Feng Qin

**Affiliations:** Department of Virology, State Key Laboratory of Pathogen and Biosecurity, Beijing Institute of Microbiology and Epidemiology, Beijing, 100071 China

**Figure 1:**
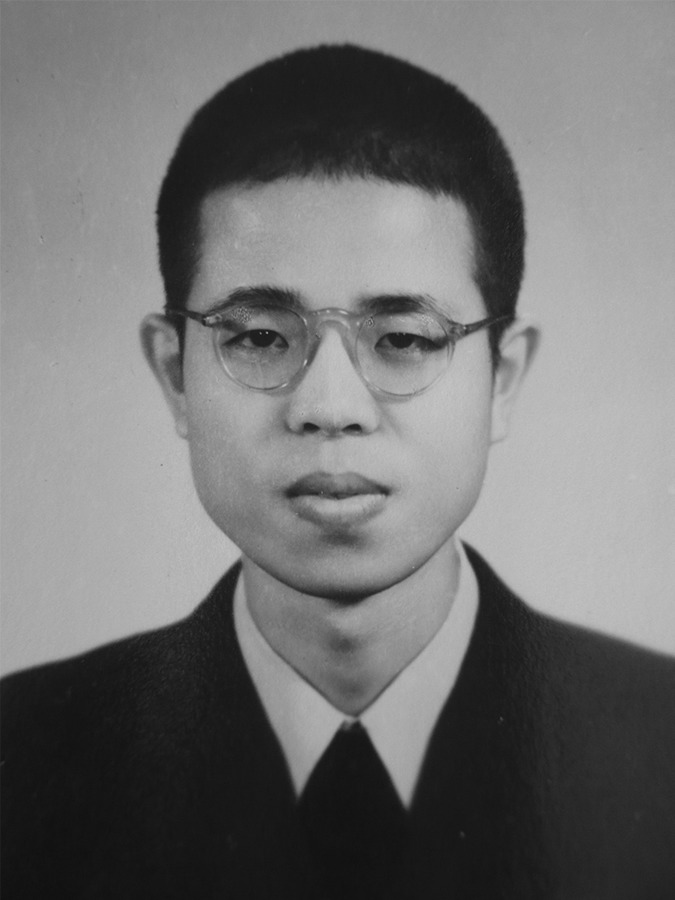
Prof. Guan-Fu Zhu in his youth

Born in Dinghai County of Zhejiang province in June, 1926, Guan-Fu Zhu began to study at School of Medicine, Tongji University, in October, 1946 (Fig. 1). Then he moved to the Department of Bacteriology and Immunology of Peking Union Medical College in September, 1954, where he got his master degree under the supervision of Prof. Shao-Wen Xie, a renowned microbiologist and immunologist. In July, 1951, Mr. Zhu served as an assistant in teaching and research section of the Second Military Medical University of PLA. In January, 1958, he began to work at the Institute of Microbiology and Epidemiology, Academy of Military Medical Sciences, successively serving as a research assistant, assistant professor, associate professor and professor. Due to cardiovascular and cerebrovascular diseases, Mr. Guan-Fu Zhu unfortunately passed away on November 7th, 2015, at the age of 89.

Prof. Zhu made significant contribution to the development of virology in China and was deemed as one of the pioneer virologists in China. In the early 1970s, Prof. Zhu successfully isolated dozens of viral strains of coronaviruses, rhinoviruses, and adenoviruses from nasal discharge and pharyngeal fluid samples from patients with cold (Huang et al., 1979; A group of cold prevention and control of PLA 1975; Zhu 1976), and investigated the infection rate of coronavirus in population, providing basic foundation for the study of the etiology of respiratory infectious diseases in China. In the early 1980s, Prof. Zhu systematically established virus propagation techniques in tissue culture including a variety of viruses such as vector borne alphavirus and flavivirus (Zhu et al., 1982; Liu et al., 1982). Then at the beginning of 1990s, he and his colleagues isolated the first Chinese HIV strain from the blood samples of a Chinese patient using co-culture of lymphocyte (Sun et al., 1991). In addition, Prof. Zhu had tried to utilize the monoclonal antibody technology to the laboratory detection and diagnosis of common viral diseases at the very early stage. These brilliant works lead to numerous publications in scientific journals and textbooks. He also co-edited several keystone monographs, including *Virus Names* (Science Press, Zhu 1987), *Modern clinical virology* (People’s Military Medical Press, Du et al., 1991), and *Prevention and Control of Common Emerging infectious diseases* (Zhejiang University Press, Shao et al., 2005).

Prof. Guan-Fu Zhu spent all his energy and life time on virology research. His personality affects everyone he meets, and his old friends called him “Old Master Zhu”. Prof. Zhu had made great contributions to the development of Chinese Society of Virology and the international communication and collaboration in the field of virology. Mr. Zhu served as three-term vice president for the council of the Chinese Society for Microbiology, and was then awarded the title of honorary chairman. Since 1984, Guan-Fu Zhu was elected as the chairman (1984–2001) of the Virology Committee of the Chinese Society for Microbiology. Prof. Zhu had launched the National Conference on Virology, held by the Virology Committee of the Chinese Society for Microbiology. It has been held biannually since the first meeting in Tianjin, China in July 15–19th, 1986, and has become a grand gathering for all the Chinese virologists. The 11th National Conference on Virology held in Wuhan in 2015 attracted more than 1200 participants. Prof. Zhu was active in organizing and participating the academic activities even after retirement, and served as the honorary chairman for the sixth session of Virology Committee and the fifth session of the Microbiology and Immunology Branch of Chinese Medical Association. In 2009, Prof. Zhu, at the age of 83, still attended the 8th National Conference on Virology in Beijing, and communicated with next generation of Chinese virologists.

**Figure 2:**
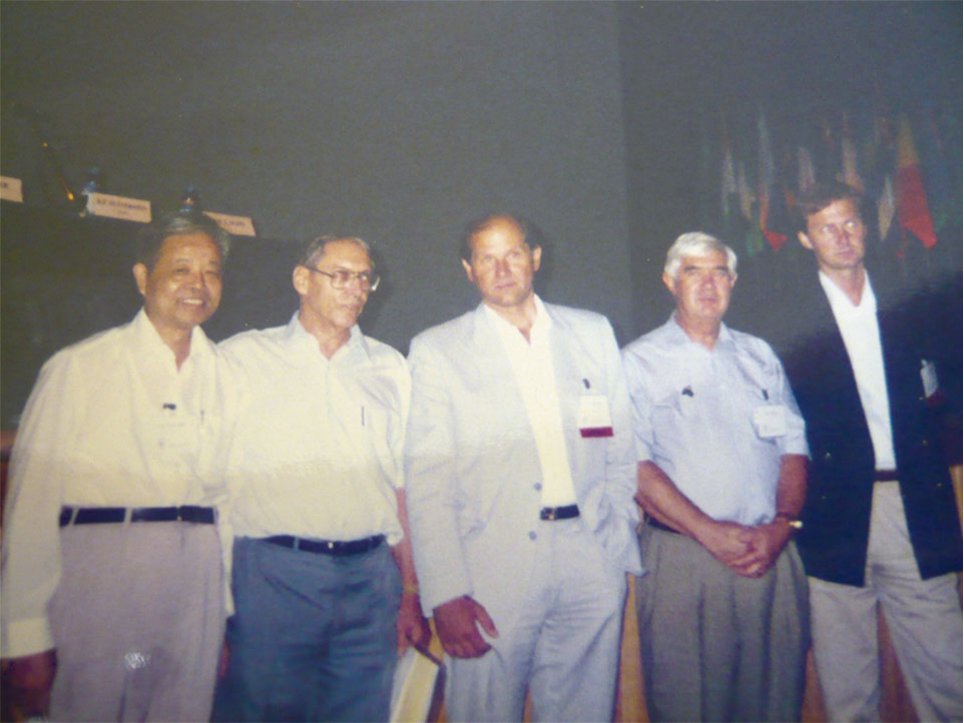
Prof. Guan-Fu Zhu (in the left) in the 10th International Conference on Virology, 1996

**Figure 3:**
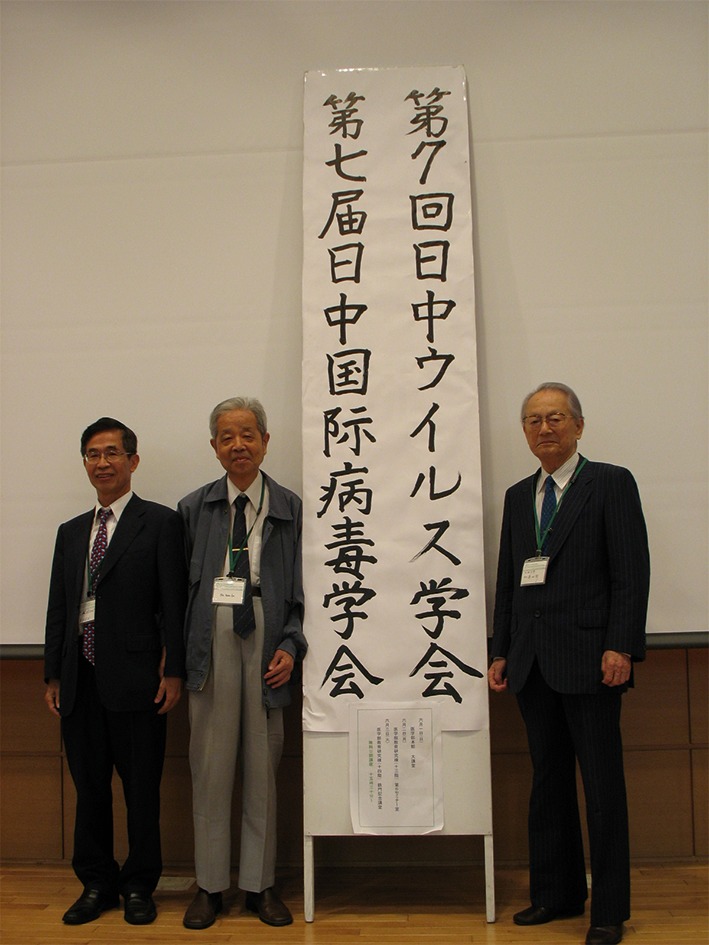
Prof. Guan-Fu Zhu (in the middle) in the 7th China-Japan International Conference of Virology

Prof. Zhu had been actively promoting the international communications in the field of virology. On August 27–31th, 1990, as the chairman of Virology Committee of the Chinese Society for Microbiology, Prof. Zhu attended the 8th International Conference on Virology in West Berlin, held by the Virology Division of International Union of Microbiological Societies (IUMS) (Fig. 2). In 1992, Prof. Zhu initiated and launched the China-Japan International Conference of Virology with Japanese scholars in Beijing Friendship Hotel, which has been held biannually until now (Fig. 3).

“Master Kong” (Confucius) has been alternately idealized as a culturally symbolic figure of China over the millennia. Prof. Guan-Fu Zhu will be remembered for his great contribution and nobility in character, and we will miss Old Master Zhu forever.
